# Supplementary material for: Post-Acute COVID-19 Effects on Diagnostic Conversion Rates And Standardized Cognitive and Motor Test Scores in a Longitudinal Study of Independent, Community-Recruited Elderly Subjects
Source: medRxiv. 2025 Oct 22:2025.10.20.25338408. Preprint. [Version 1] doi: 10.1101/2025.10.20.25338408 (PMC12633594; doi:10.1101/2025.10.20.25338408)
Supplement: Supplement 2 [file media-2.pdf]

#28338388.4

IRB Approved at the
Study Level
Dec 07, 2021

Brain and Body Donation Program Covid-19 Medical History Research Questionnaire

Version 5, 11/18/2021; Thomas G. Beach, MD, PhD

Introduce to participant as: "This questionnaire is to gather information about your experience with Covid-19, also known as Coronavirus, also known as SARS or SARS- CoV-2".

Date of Questionnaire Administration/Interviewer Name: ____

BBDP Participant Name/DOB/Donor ID: ____

BBDP Study Partner Name/DOB (month /year only): ____

Person Completing Questionnaire (circle all that apply): Participant Study Partner or Relative BBDP Personnel

Questionnaire Supplemented by Medical Records Review (circle): Yes No Deferred

Have you had Covid-19, or an illness caused by coronavirus (circle): Yes No Not Sure

Has a healthcare provider ever told you that you had Covid-19 (circle) Yes No Not Sure
If Yes, please provide healthcare provider's name and phone number: ___________________________________

Have you ever been tested by nose swab for Covid-19 (circle): Yes No or Not Sure
If Yes, what was the approximate date of the test? Exact Date_____________or Month/Year ______________
If Yes, what was the test result (circle): Positive Negative Indeterminate or Not Sure

Have you ever had a blood test for Covid-19 (circle): Yes No or Not Sure
If Yes, what was the approximate date of the test? Exact Date_____________or Month/Year _______________
If Yes, what was the test result (circle): Positive Negative Indeterminate or Not Sure

Where was Covid-19 testing done (circle all that apply): Doctor's Office Urgent Care Medical Lab Hospital Drive-Up

Did you ever go to a hospital because of Covid-19? Yes No or Not Sure

If Yes, what hospital: _______________________

If Yes, Exact Dates________________or Month/Year ______________

If Yes, what doctor/provider took care of you there: _____________________________________ or Not Sure

Where in the hospital were you (circle all that apply): Emergency Inpatient ICU Covid-19 area or Not Sure

What breathing treatment did you have (circle all that apply): None Oxygen Ventilator ECMO or Not Sure

What medicines were you treated with (circle all that apply): or Not Sure

Azithromycin Other antibiotics Dexamethasone Inhaled Corticosteroids (flovent, symbicort, Advair)

Oral corticosteroids (prednisone) Remdesivir Convalescent Serum Hydroxychloroquine/Choroquine

Anti-coagulants (blood thinners) Monoclonal antibody Pfizer Covid pill (Paxlovid)

Merck Covid pill (molnupiravir)

How long were you in the hospital (circle): Few Days Week or More Month or More or Not Sure

Did participant die as a result of Covid-19 illness? Yes No

Have you been having, or having with increased frequency or severity (relative to previous years) of any of these symptoms over the past year? (circle all that apply):

Fever Chills Shakes Muscle Aches Runny Nose Sore Throat Loss of Taste Loss of Smell

Headache Fatigue Difficulty Concentrating Cough Wheezing Shortness of Breath/Difficulty Breathing Chest Pain Nausea Vomiting Abdominal Pain Diarrhea Stroke Transient Ischemic Attack (TIA)
Myocardial Infarction (Heart Attack) Kidney Failure

Have you ever been vaccinated or immunized for Covid-19 or coronavirus? (circle) Yes No or Not Sure

If yes, what type of vaccine was it? (circle one of the choices below):

Pfizer Moderna Astra-Zeneca Johnson & Johnson Janssen Novavax or Not Sure

If Yes, what was the approximate date of the vaccination? Exact Date ___________or Month/Year __________

If the vaccination required a second dose, give date here. Exact Date ___________or Month/Year __________

Have you ever had a booster vaccination for Covid-19 or coronavirus? (circle) Yes No or Not Sure

If yes, what type of vaccine was it? (circle one of the choices below):

Pfizer Moderna Astra-Zeneca Johnson & Johnson Janssen Novavax or Not Sure

If Yes, what was the approximate date of the vaccination? Exact Date ___________or Month/Year __________
